# Infant-directed speech facilitates seven-month-old infants’ cortical tracking of speech

**DOI:** 10.1038/s41598-018-32150-6

**Published:** 2018-09-13

**Authors:** Marina Kalashnikova, Varghese Peter, Giovanni M. Di Liberto, Edmund C. Lalor, Denis Burnham

**Affiliations:** 1 The MARCS Institute for Brain, Behaviour and Development, Western Sydney University, Locked Bag 1797, Penrith, 2527 Australia; 2 School of Engineering, Trinity Centre for Bioengineering, and Trinity College Institute of Neuroscience, Trinity College Dublin, Dublin, Ireland; 3 Laboratoire des Systèmes Perceptifs, Ecole Normale Supérieure, Paris, 75005 France; 4 Department of Biomedical Engineering and Department of Neuroscience, University of Rochester, Rochester, New York, 14627 USA

## Abstract

This study assessed cortical tracking of temporal information in incoming natural speech in seven-month-old infants. Cortical tracking refers to the process by which neural activity follows the dynamic patterns of the speech input. In adults, it has been shown to involve attentional mechanisms and to facilitate effective speech encoding. However, in infants, cortical tracking or its effects on speech processing have not been investigated. This study measured cortical tracking of speech in infants and, given the involvement of attentional mechanisms in this process, cortical tracking of both infant-directed speech (IDS), which is highly attractive to infants, and the less captivating adult-directed speech (ADS), were compared. IDS is the speech register parents use when addressing young infants. In comparison to ADS, it is characterised by several acoustic qualities that capture infants’ attention to linguistic input and assist language learning. Seven-month-old infants’ cortical responses were recorded via electroencephalography as they listened to IDS or ADS recordings. Results showed stronger low-frequency cortical tracking of the speech envelope in IDS than in ADS. This suggests that IDS has a privileged status in facilitating successful cortical tracking of incoming speech which may, in turn, augment infants’ early speech processing and even later language development.

## Introduction

Cortical tracking refers to the process by which cortical activity tracks dynamic patterns of incoming information, in this case speech input. This applies to both low-level spectrotemporal speech features as well as higher-level speech-specific information^1–5^. The speech envelope contains linguistic information across multiple time scales: at the phonological rate (about 30–50 Hz, corresponding to the gamma band of neural oscillations at which information such as place of articulation, e.g., /b/ vs. /d/ and voicing, /p/ vs. /b/ is conveyed); at the syllabic rate (about 4–8 Hz, corresponding to the theta band of neural oscillations); and at the lexical and phrasal rate (<2 Hz, within the delta band of neural oscillations). Simultaneous cortical tracking of the speech signal across these different temporal scales allows sampling of the incoming speech stream during speech processing^1,6^.

Neurophysiological indices of cortical tracking of natural speech can be successfully extracted from electroencephalography (EEG) or magnetoencephalography (MEG) recordings in adult and child participants. Importantly, accurate tracking of the speech envelope has been shown to be affected by changes in intelligibility^7,8^ and by mechanisms such as multisensory integration^9^. In addition, selective attention^6,10–12^ has been shown to play a significant role in facilitating cortical tracking as demonstrated when adults and school-aged children are presented with different strings of speech input to each ear and asked to switch their attention from one string to the other^3,6,10–12^. In such studies, recordings of neural activity at the theta band (syllabic rate) using MEG show that neural activity is correlated with the acoustic amplitude envelope of both the attended and unattended streams, but that the patterns of correlation differ for both streams as a function of attention, with more accurate tracking recorded in response to the attended stream.

These findings provide strong evidence that cortical tracking involves attentional mechanisms, enhancing listeners’ ability to focus selectively on a single speech stream and to filter out other competing auditory information (known as the ‘cocktail party’ effect). However, these studies have focused solely on adults and school-aged children – proficient language users whose extensive phonetic, syntactic, and semantic linguistic competence facilitates processes of parsing and encoding natural speech; and who can be asked to direct their attention to a particular speech stream from several competing speech streams. This is not the case for young infants. Therefore, we aim to tackle two core questions in this study: whether cortical tracking of speech can be measured in young preverbal infants, and if so, whether it is responsive to or even facilitated by augmented attention-grabbing speech. The first question is relevant from a methodological perspective as we propose to evaluate the validity of a measure with infants that has previously been used only with older participants. Furthermore, the answer to this first question is theoretically important, for if there is such cortical tracking of speech, then it would mean that preverbal infants’ endogenous neural oscillations entrain to incoming speech before any extensive knowledge of the phonetic, semantic, and syntactic properties of their native language is acquired. The second question is also theoretically important: if infants’ cortical tracking is affected by the attention-grabbing properties of speech input, then it would imply that infant directed speech, the type of speech input to which infants are exposed in daily interactions with their parents, may facilitate processes of cortical tracking of speech and early speech processing.

While evidence of cortical tracking processes early in life is limited, Telkemeyer and colleagues^13^ have shown that the newborn brain is already sensitive to the temporal structure of speech. They presented newborns with frequency-modulated non-speech stimuli corresponding to the phonological and the lexical speech rates and recorded their electrophysiological and haemodynamic responses that were considered to be equivalent to the adult auditory steady-state response (ASSR), which is phase-locked to the amplitude envelope of auditory input. Infants’ neural responses were shown to be tuned to these non-speech modulations, and tuned similarly for the analogues of the phonemic and lexical speech rates. However, to date, cortical tracking of *continuous natural speech* has not been assessed in infants. This is the first aim of this study.

The second aim of this study is to investigate whether infants’ cortical tracking is facilitated by what we know to be attentionally-salient to infants, infant-directed speech. Young infants are exposed to extensive linguistic input, both directed to them, infant-directed speech (IDS), and directed to adults around them, adult-directed speech (ADS). These two speech registers differ markedly: compared to ADS, IDS is characterised by slower tempo and speech rate^14^, regularised rhythm^15–17^, higher emotional content^18^, higher pitch and greater pitch range^19^, simplified grammatical structure^20^, and acoustic exaggeration of speech sounds^21–23^. Given its greater capacity to capture infants’ attention, IDS may afford better opportunities for cortical tracking of incoming speech in young infants than might ADS. There are two lines of evidence that provide traction for this possibility.

First, infants prefer IDS to ADS (see^24^ for a review). This preference is present in newborns^25^ even when IDS is produced by unfamiliar females or males^19^ or in a foreign language^26^. Thus, it appears that preference for IDS is driven by its general acoustic and prosodic qualities rather than any particular indexical characteristics. This is further evidenced by neurophysiological studies showing greater cortical activity in temporal and frontal sites for infants up to 12 months of age in response to naturally-produced IDS compared to ADS (functional near-infrared spectroscopy^27,28^; electroencephalography^29^).

Second, exposure to IDS appears to facilitate linguistic processing during the child’s first years of life. Behavioural and neurophysiological studies indicate that IDS is better than ADS in promoting performance on a variety of linguistic tasks such as speech sound discrimination^30,31^, familiar word recognition^32,33^, and word learning^34,35^. Thus, it is possible that by using IDS, parents unconsciously produce not only the type of speech that their infants attend to and prefer, but also the type of speech that assists their infants in the challenging task of learning their native language^36^.

While the mechanisms by which IDS might facilitate early language development remain unspecified^37^, it may just be that IDS facilitates the cortical tracking of speech, which in turn might underlie enhanced performance in the abovementioned linguistic tasks. In this study, we investigate (i) pre-verbal infants’ processing of continuous speech by measuring the tracking of incoming speech by cortical activity; and (ii) whether IDS has a privileged role in facilitating the process of cortical tracking compared to ADS. Our recent research provides a framework for investigating cortical tracking of continuous speech features, e.g., amplitude envelope and phonetic distinctions, using non-invasive EEG^2,38,39^. The approach is based on a ridge regression fit between the speech envelope and the EEG signal and allows prediction of the resulting EEG (forward-modelling), the reconstruction of the speech envelope (backward-modelling), and the derivation of quantitative measures that have been linked to cortical entrainment^9,12,40^. The regression model weights (temporal response functions, TRFs) can be studied in terms of their spatio-temporal dynamics in a similar manner to event related potentials (ERPs^9^), with the important difference that continuous natural stimuli can be used. In particular, this includes examining the distribution of the TRF weights across the scalp at different latencies, that is at different relative time lags between the ongoing speech signal and the ongoing EEG signal. For example, a latency of 100 msec means the impact that a change in the stimulus at time *t* has on the EEG at time *t* + 100 msec.

This is the first study to use this method with preverbal infants. Here, the level of coupling between infants’ cortical activity and the speech envelope in naturally-produced IDS vs. ADS is indexed by relative TRF fit and the relative accuracy of prediction of the unseen EEG signal based on the envelope of the speech signal. There are two specific predictions. First, to confirm that IDS is more attentionally-salient than ADS^25^, we expected greater frontal EEG power to IDS than to ADS measured in cortical responses at the theta band^29^. Second, we predicted that, similar to adult and school-age children, infants’ cortical tracking of the speech stimuli will be indexed by a correlation between their cortical responses and the low-level acoustic envelope of the speech signal. However, given the differences in attentional salience between IDS and ADS, we expected the envelope to be more strongly reflected in the EEG responses to IDS than ADS.

## Results

### EEG Power Analysis

**Figure 1:**
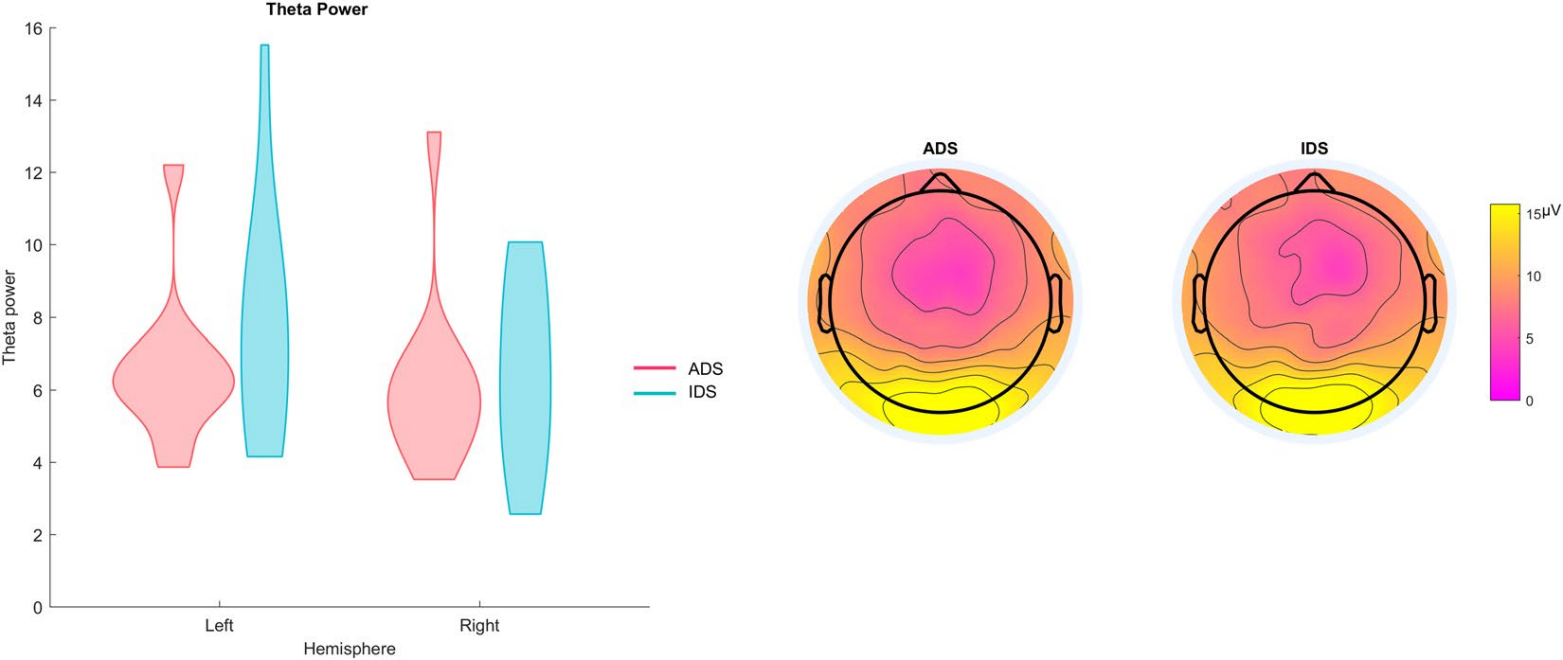
Results of the EEG power analysis averaged for all epochs recorded in IDS and ADS (the left panel displays the EEG power distribution in the theta-band range (4–8 Hz) for IDS and ADS across hemispheres and the right panel displays the scalp topography of the Mean theta power).

Amplitude of the EEG power distribution in the theta-band range (4–8 Hz) across the left and right hemispheres is shown in Fig. 1. A two-way Analysis of Variance (ANOVAs) with register (ADS, IDS) and hemisphere (left, right) as the within-subjects factors and theta power as the dependent variable showed a main effect of hemisphere, *F*(1,11) = 10.15, *p* = 0.001, *η*^2^ = 0.48, indicating that the theta power over the left hemisphere (*M* = 7.14, *SE* = 0.73) was significantly larger than over the right hemisphere (*M* = 6.28, *SE* = 0.64). Neither the main effect of register, *F*(1,11) = 2.28, *p = *0.16, *η*^2^ = 0.17, nor the interaction between register and hemisphere, *F*(1,11) = 2.26, *p* = 0.16, *η*^2^ = 0.16, were significant. Therefore, contrary to our prediction, theta power did not differ between the IDS and ADS conditions, only between hemispheres, irrespective of register.

### TRF Analysis

**Figure 2:**
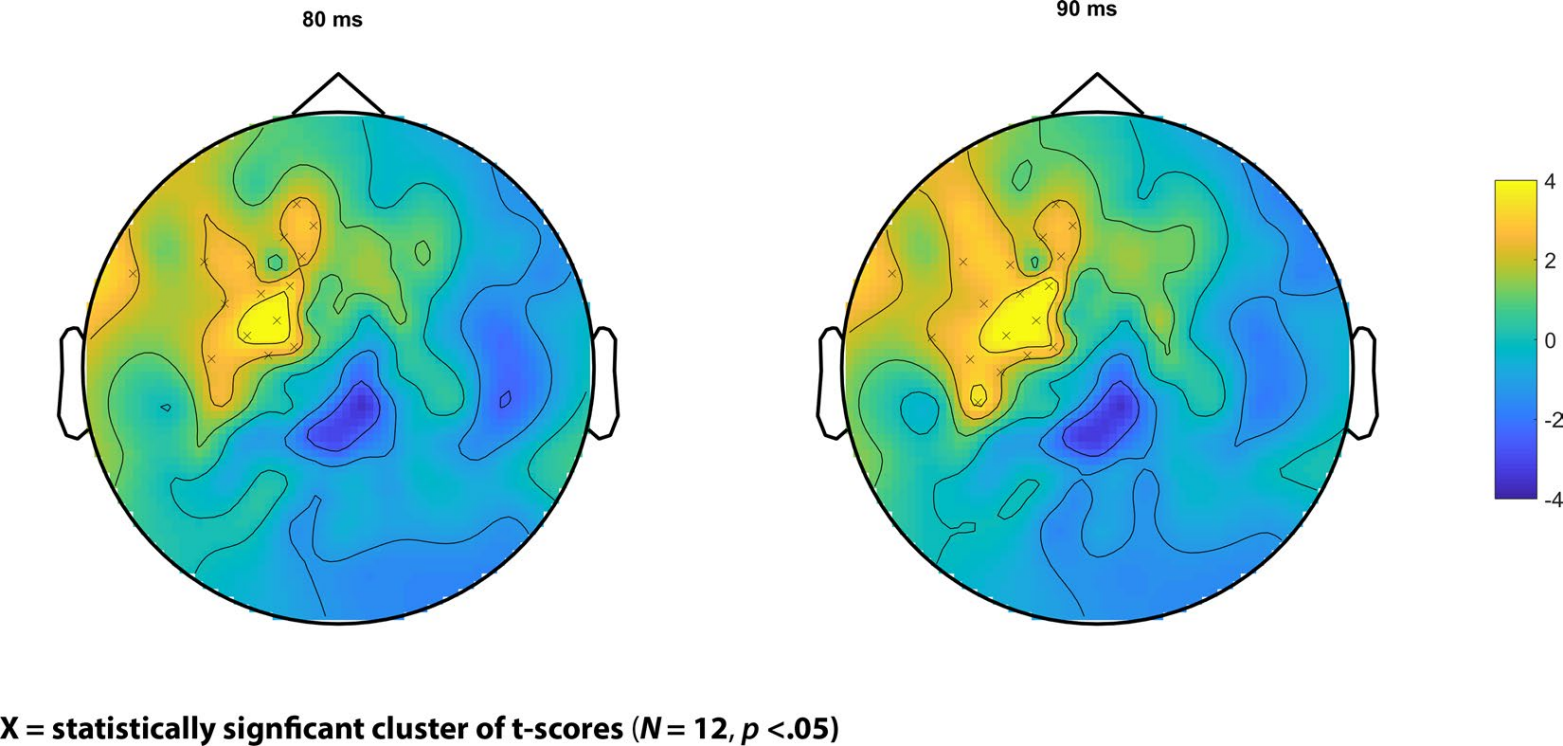
Results of cluster permutation statistics on the TRFs for IDS and ADS registers.

In order to detect any difference between TRFs for the IDS and ADS registers as a function of speech-EEG time lag, we used a cluster-based permutation analysis^40^. This allows for the identification of clusters of electrodes in which significant response differences between the two registers are detected while controlling for Type I error that may arise due to multiple tests conducted for each electrode (see Method section). The results of this analysis revealed significant differences between the TRFs for IDS and ADS registers in the 80–90 msec time range. Over the left hemisphere, IDS had a significant positive response between 80–90 msec as compared to ADS (Cluster *p* = 0.046). The location of this cluster for each time point is indicated in Fig. 2.

### Cortical Entrainment Analysis

**Figure 3:**
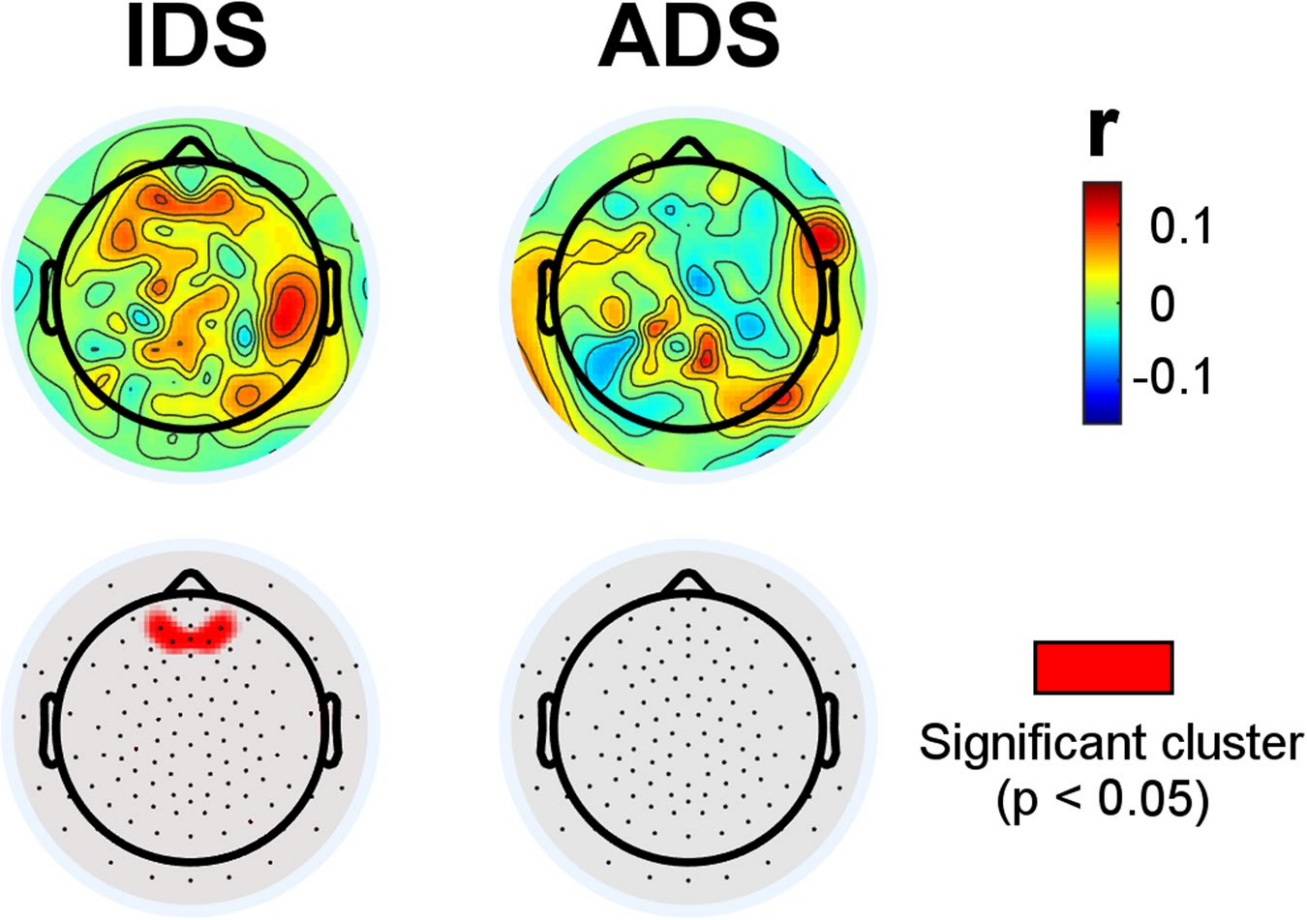
EEG prediction correlations for the IDS and ADS conditions (the top panel shows the correlation *rho* values and the bottom panel highlights the electrodes for which the *rho* values are significantly different from zero).

The TRFs obtained for ADS and IDS were used to predict the EEG signal using leave-one-out cross-validation^2,38^. Specifically, Pearson’s correlation values between the recorded EEG and its prediction were used to index sensor-space cortical tracking of the speech envelope at the individual subject-level. This results in a distribution of correlation values, one for each subject and electrode, that is used to identify significant clusters of scalp electrodes that consistently track the speech envelope. Here, as depicted in Fig. 3, significant prediction correlations emerged in response to IDS, forming a cluster composed of 8 electrodes in the frontal area (*p = *0.023), but this frontal cluster did not emerge in response to ADS. Note that both IDS and ADS showed clusters of electrodes on the right hemisphere that were trending toward statistical significance (*p* ≃ 0.1) but were excluded after the correction for multiple comparisons.

## Discussion

This study provides the first evidence for neural tracking of naturally-produced continuous speech by preverbal infants. Neural tracking of continuous speech is associated with effective speech perception^4^. Accordingly, the facility to investigate such mechanisms non-invasively adds a powerful tool to the study of continuous natural speech^3^, complementing the traditional event-related approach which is typically used to measure cortical responses to isolated sounds (e.g., syllables, words). In this sense, the TRF approach is an effective method for quantifying cortical tracking of speech sounds using non-invasive EEG^9^, and one that is sensitive to the effects of selective attention and multisensory integration^9,38^. Our findings demonstrate that when seven-month-old infants listened to IDS and ADS, there were no significant differences between the theta band power distribution in response to the two registers, but there were significant correlations between infants’ cortical activity patterns and the envelope of the speech signal for IDS but not for ADS.

The TRFs were generally larger over the left hemisphere, and they were especially so for IDS than for ADS. This is interesting as the speech envelope was filtered between 1–8 Hz, and according to the asymmetric sampling in time (AST) theory^41^, left and right auditory cortices show oscillations at different preferred rates: gamma (25–45 Hz) in the left hemisphere and delta-theta (1–7 Hz) in the right. This left-right asymmetry has been observed regardless of the nature of the stimuli (speech or non-speech) and regardless of the involvement of higher-level speech processing^42,43^. Despite this fact that the left hemisphere is associated with rapid temporal processing^44^, our finding aligns with previous studies showing that low frequency information is processed in the left auditory cortex in young pre-verbal infants^45,46^. These findings suggest that the left hemisphere plays a major role in the early processing of low frequency amplitude envelopes. Specifically, increases in left- and front-localised theta oscillations have been proposed to represent the encoding of segmental information in speech^46^ as well as greater general attention to speech stimuli in young infants^47^.

The human perceptual system is exposed to multiple sources of information at any given time, and the ability to entrain selectively to one speech stream may be crucial in real-time decoding of information at the three levels of the speech processing (e.g., phoneme, syllable, lexical). This may be even more complex in young infants whose native language competence is still being acquired. The findings here indicate that such acquisition may be facilitated by IDS. That is, IDS is an enriched speech register that augments infants’ encoding and decoding of speech.

Previous research has shown that infants’ performance on a number of language processing tasks is enhanced when stimuli are presented in IDS compared to ADS. The results of this study add neural tracking of natural speech to the list of processes that are facilitated by speech input via IDS. Nevertheless, it is unclear from these findings what specific characteristics of IDS lead to the processing benefits attributed to this register. Two possibilities suggest themselves. First, as stated in the predictions of this study, these findings may be due to top-down processes such as greater attention elicited by the prosodic properties of IDS. This conclusion dovetails with previous research with adult participants in attention-based paradigms^40^, and the evidence that IDS attracts greater attentional responses in infants^25,27–29^. This explanation can be further strengthened by the fact that our analyses compared neural tracking of two speech signals, IDS and ADS, instead of a silence baseline^29^ or a non-speech condition, e.g., amplitude modulated noise^13^, indicating that the general attention-grabbing qualities of IDS rather than specific linguistic information underlie the difference between the two registers uncovered here.

The second possibility is that bottom-up processes play a role in facilitating cortical tracking in IDS, as IDS is also distinguished from ADS on characteristics such as phonetic realisation^21^, greater pitch range^48^, and regularised speech rhythm^16^. This view has been proposed in previous literature whereby specific components of IDS have been identified in relation to individual linguistic tasks. For instance, exaggerated productions of phonemes in IDS have been related to better discrimination of native speech sounds^49^, exaggerated prosodic patterns have been proposed to facilitate continuous speech segmentation^50^, and the vowel hyperarticulation and speech rate components of IDS have been shown to facilitate lexical processing^33^. The two possibilities proposed here are of course not mutually exclusive. It is plausible that all these prosodic and linguistic components of IDS act in unison: greater correlation between the stimulus amplitude envelope and the neural activity envelope recorded with EEG may be a product of infants’ tendency to direct their attention to IDS, and also of the encoding of individual features of the incoming speech string.

Tracking of the acoustic envelope of incoming speech by the endogenous neural oscillations has been linked to successful encoding of auditory speech. This occurs when adult listeners selectively attend to the speech input in their environment^40^. This study demonstrates that a similar process occurs when infants as young as seven months of age listen to speech. Most importantly, there is a greater correlation between neural activity and the speech envelope when infants listen to IDS compared to ADS. These findings suggest that the special register that parents spontaneously use when addressing their young infants (and which has been found to foster early linguistic processing) facilitates speech encoding at its multiple timescales even at the earliest stages of language acquisition. Further work is required to locate the precise source of this early difference in processing IDS and ADS input as well as to identify the developmental time course for neural tracking of ADS.

## Method

### Participants

Twelve seven-month-old infants participated in the experiment (6 female; *M* age = 225.1 days, *SD* = 9.1). All infants were acquiring English as their first language, were born full-term, and were not at-risk for cognitive or language delay. Seven additional infants were tested, but their data were removed from the analysis as five infants had more than 20 bad channels and two infants did not complete the experiment. This study was approved by the Human Research Ethics Committee at Western Sydney University (approval number 9142). Prior to the study, the primary caregiver of each infant completed an informed consent form, and they were informed that the procedure would be immediately discontinued if they wished so, or if their infant showed any signs of distress. This study followed the approved protocol regarding participant recruitment, data collection, and data management.

### Stimuli

The stimuli consisted of recordings of naturally-produced IDS and ADS. These recordings were produced by a female Australian English speaker when she interacted with her seven-month-old infant (IDS) or an experimenter (ADS). During the IDS recording, the speaker and her infant sat alone in an infant laboratory room, and she was instructed to interact naturally with her baby. She was provided with soft toys and pictures to facilitate the interaction. During the ADS recording, the speaker was interviewed by a female experimenter, also a native speaker of Australian English, in the same laboratory room and she was asked to comment about the IDS session. The infant was not present during the ADS recording. A head-mounted microphone (AudioTechnica AT892) connected to Adobe Audition CS6 software via an audio input/output device (MOTU Ultralite MK3) was used during the speech recordings.

The ADS recording was 481 seconds in duration and the IDS recording was 486 seconds in duration. As expected, compared to ADS, IDS had higher pitch and greater pitch range (IDS: *M* F0 = 202.19 Hz, F0 range = 385.88 Hz; ADS: *M* F0 = 171.53 Hz, F0 range = 298.59 Hz), slower speech rate (IDS: 2.85 words/second, 1384 words in total; ADS: 3.76 words/second, 1810 words in total), and hyperarticulated vowels (based on the area of the triangle resulting from plotting F1 and F2 values for the three corner vowels /i/, /u/, and /a/; IDS area = 17263.26 Hz^2^, ADS area = 9455.50 Hz^2^).

### EEG Recording

The infants sat on their parent’s lap approximately 1 m from an LCD screen. Stimuli were presented through audio speakers at 75 dB SPL. In order to maintain infants’ attention, a coloured checkerboard was presented on the screen which changed colour every 30 seconds. While infants listened to the speech stimuli, their continuous EEG was recorded using a 129 channel Hydrocel Geodesic Sensor Net (HCGSN), NetAmps 300 amplifier and NetStation 4.5.7 software (EGI Inc) at a sampling rate of 1000 Hz with the reference electrode placed at Cz. The electrode impedances were kept below 50 kΩ. The continuous EEG was saved for offline analysis.

### EEG Pre-processing

The EEG analysis was performed using EEGLAB^51^, FieldTrip^52^, the mTRF toolbox^38^, and custom scripts in MATLAB2014a^53^. Since the infant EEG recordings are noisy due to infant movements, we applied artifact subspace reconstruction (ASR^54^) to remove noise. ASR uses a sliding window technique whereby each window of EEG data is decomposed via principal component analysis so it can be compared statistically with data from a calibration dataset. Within each sliding window the ASR algorithm identifies principal subspaces, which significantly deviate from the baseline EEG and then reconstructs these subspaces using a mixing matrix computed from the baseline EEG recording. In this study, we used a sliding window of 500 msec and a threshold of 20 standard deviations to identify corrupted subspaces. The noisy channels that were removed during the ASR procedure were later replaced by averaging the neighbouring channels weighted by distance. The EEG was then analysed in two ways: EEG power analysis and temporal response function (TRF) analysis.

### EEG Power Analysis

First EEG data were down-sampled to 250 Hz for computational reasons. The EEG was then divided into one second non-overlapping epochs starting from the onset of the ADS and IDS recordings. EEG epochs with amplitude fluctuations exceeding ±100 µV were removed. The data were then re-referenced to the average of all the electrodes. All participants had at least 100 artifact-free epochs (corresponding to 100 seconds of EEG data; ADS *M* = 206.5, *SD* = 25.23; IDS *M* = 203.52, *SD* = 31.55). All artifact-free EEG data were analysed using a discrete Fourier transform (DFT), with a Hanning window of one second width and 50% overlap. Power (µV^2^) was derived from the DFT output in the 4–8 Hz frequency band for ADS and IDS conditions.

EEG power in the theta region was then computed across two scalp locations: frontal left and frontal right. Frontal electrodes were selected for the analysis as auditory stimuli generate larger responses at frontal electrodes (see the Supplementary Fig. S1 for a graphical representation of these electrode groupings).

### TRF Analysis

The data were first re-referenced to average reference. The amplitude envelope of speech between 1–8 Hz was then extracted using Hilbert transform. In order to reduce the computational time, both speech envelope and EEG were down-sampled to a sampling rate of 128 Hz. Twenty-one electrodes on the periphery of the electrode net were removed as it generally records noisy data in infants, and it is common to remove these peripheral electrodes when conducting the analysis of infant EEG^55,56^.

A ridge regression model was fitted from the speech envelope to the EEG signal for every participant and channel^38^. The window was restricted to the 0 msec to 500 msec window because no visible response emerged out of that lag interval. The regularisation parameter of the model (lambda) was chosen using a quantitative procedure that aims at producing an optimal model fit. A lambda of 1 was selected for the current analysis. The resulting regression weights, referred to as temporal response functions (TRFs), were studied in terms of their spatial and temporal dynamics, similar to an ERP analysis. The TRFs obtained from individual participants were averaged to obtain the grand averaged TRF waveform.

**Figure 4:**
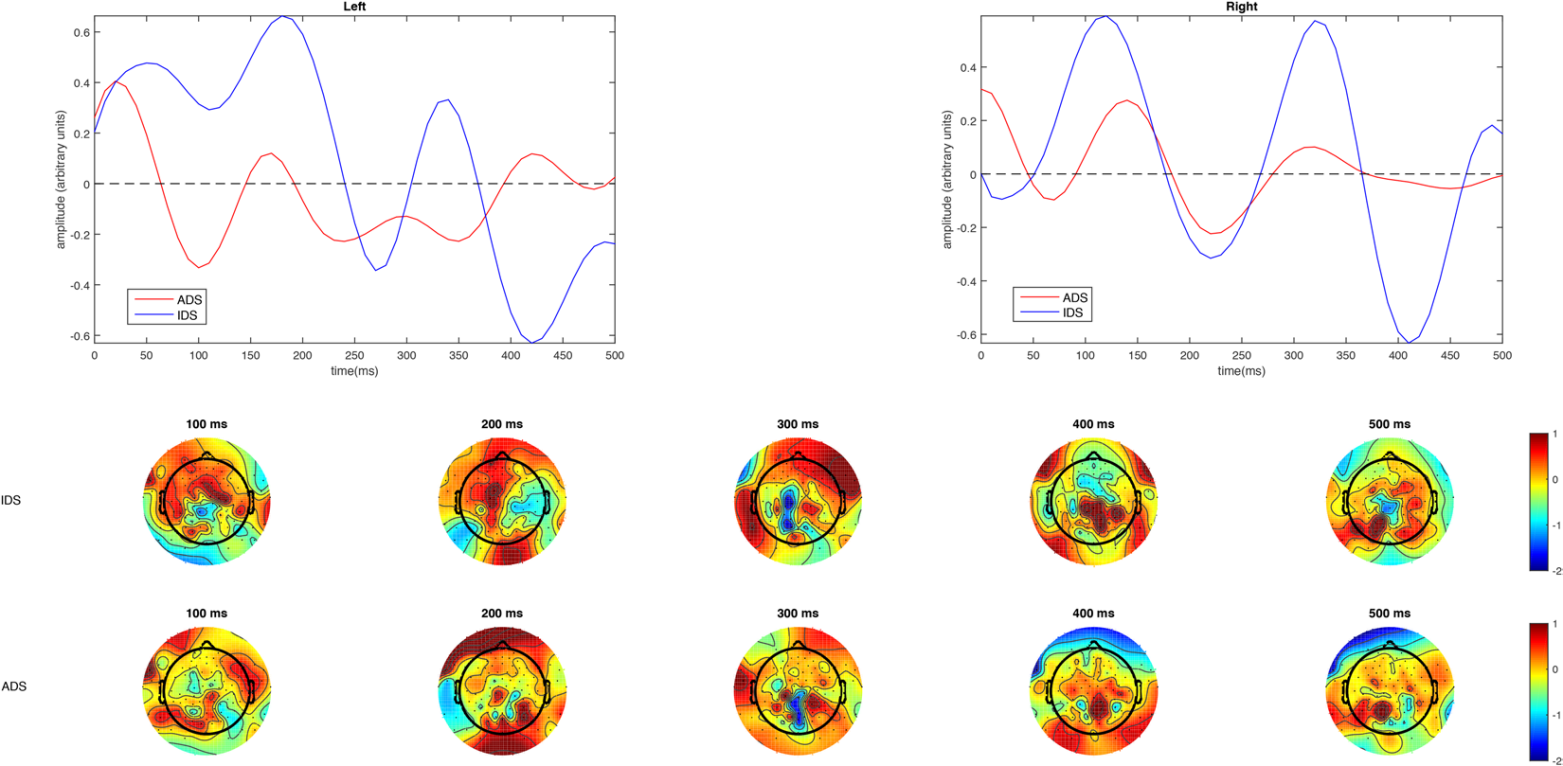
Temporal Response Function (TRF) analysis for IDS and ADS registers (the top panel displays the TRFs for ADS and IDS over the left and right hemispheres, and the bottom panel displays the scalp topography of the TRFs from 100 to 500 msec).

Five-fold leave-one-out cross-validation was used to assess how well the unseen EEG data could be predicted. This was achieved by using the TRF fit on 4 folds to predict the EEG data of the 5^th^ fold, and to iterate this procedure for all combinations. If EEG can be predicted with accuracy significantly greater than zero, it can be asserted that the EEG is reflecting the encoding of the envelope of speech^38^. Prediction accuracy was measured by calculating Pearson’s (*r*) linear correlation coefficient between the predicted and original EEG responses at each electrode channel. The time window that best captures the stimulus-response mapping is used for EEG prediction (i.e., *T*_min_, *T*_max_). This is identified by examining the TRFs on a broad time window (e.g., −100 msec to 600 msec) and then choosing the temporal region of the TRF that includes all relevant components that map the stimulus to the EEG with no evident response outside of this range. In this case, the time window chosen for this quantitative analysis was 0–500 msec. Figure 4 shows the TRFs as well as the scalp topography of the TRF peaks for ADS and IDS for this time window.

### Cluster Permutation Analysis

In order to assess the difference between TRFs for the IDS and ADS conditions at any time point of the recording, a cluster-based permutation analysis was employed^57^. In this analysis, multiple *t*-tests are computed at every electrode and every time point. From this analysis, clusters of electrodes and time points in which the response significantly differs between conditions are identified. These clusters are then formed over space by grouping electrodes that have significant initial *t*-test values at the same time point. The sum of all *t*-scores within each cluster provides a cluster-level *t*-score (mass *t*-score). A permutation approach is then used to control for Type I errors, by randomly assigning conditions and repeating the multiple *t*-tests (1000 iterations) in order to build a data-driven null hypothesis distribution. The relative location of each observed real cluster mass *t*-score within the null hypothesis distribution indicates how probable such a score would be if the null hypothesis were true. The significance of a cluster is determined by whether it falls in the highest or the lowest 2.5^th^ percentile of the corresponding distribution.

All data and tools used for analyses in this study are available upon request to the first author.

## Electronic supplementary material

Supplementary Information
