# Supplementary material for: Infant-directed speech facilitates seven-month-old infants’ cortical tracking of speech
Source: Sci Rep. 2018 Sep 13;8:13745. doi: 10.1038/s41598-018-32150-6 (PMC6137049; doi:10.1038/s41598-018-32150-6)
Supplement: Supplementary file 1 — Supplementary Information [file 41598_2018_32150_MOESM1_ESM.docx]

**Infant-directed speech facilitates seven-month-old infants’ cortical tracking of speech**

Marina Kalashnikova^1*^, Varghese Peter^1^, Giovanni M. Di Liberto^2,3^, Edmund C. Lalor^2,4^, Denis Burnham^1^

**Author affiliation:**

^1^The MARCS Institute for Brain, Behaviour and Development, Western Sydney University, Locked Bag 1797, Penrith 2527, Australia.

^2^School of Engineering, Trinity Centre for Bioengineering, and Trinity College Institute of Neuroscience, Trinity College Dublin, Dublin, Ireland.

^3^Laboratoire des Systèmes Perceptifs, Ecole Normale Supérieure, Paris 75005, France.

^4^Department of Biomedical Engineering and Department of Neuroscience, University of Rochester, Rochester, New York 14627, USA.

*Correspondence to: [m.kalashnikova@westernsydney.edu.au](mailto:m.kalashnikova@westernsydney.edu.au); +61 9772 6264

**Supplementary Information**


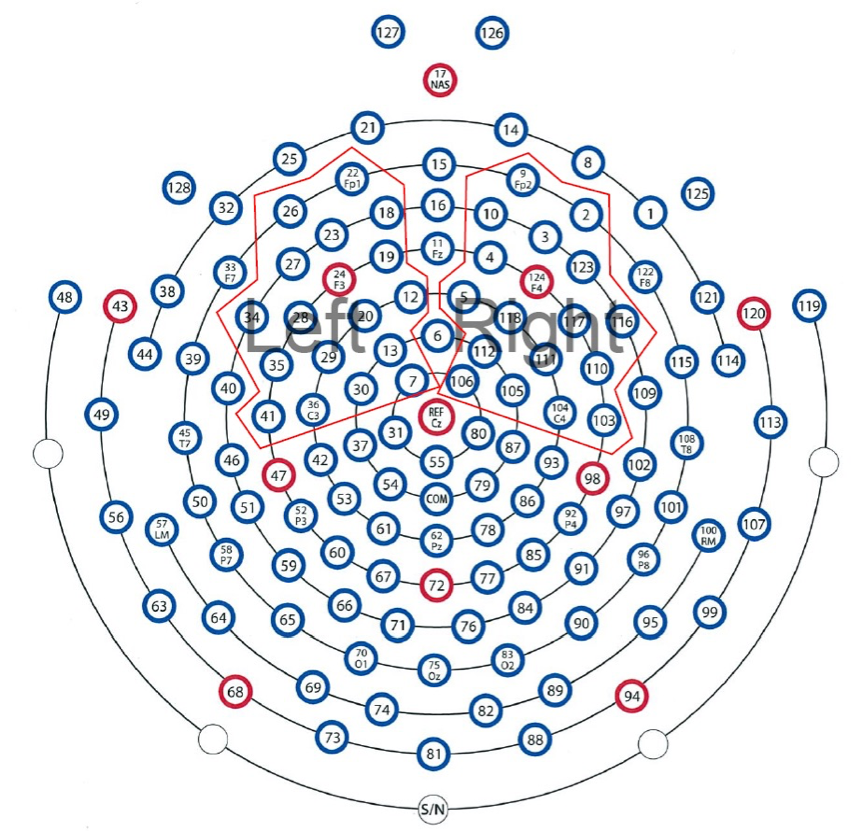


Figure SI 1. *Electrode groupings used for EEG power analysis.*
